# Antidotes of Corrosive Sublimate, Copper, Lead and Arsenic

**Published:** 1844-11

**Authors:** 


*Antidotes of Corrosive Sublimate, Copper, Lead, and Arsenic.*—By MM. Bouchardat and Sandras.—By means of numerous experiments, first made in the laboratory and then repeated on dogs, MM. Bouchardat and Sandras have arrived at many interesting results relative to the antidotes of corrosive sublimate, copper, lead, and arsenic. All these are detailed at length in their long papers on the subject. Their conclusions were, that the following substances may be regarded as antidotes, and employed as such in medicine:—

*As antidotes for corrosive sublimate*—A mixture of zinc and iron filings; or powder of iron reduced by hydrogen; or the moist persulphuret of the hydrated peroxide of iron.

*As antidotes for copper.*—A mixture of zinc and iron filings; iron reduced by hydrogen; porphyrized iron; zinc filings; or the persulphuret oi the hydrated peroxide of iron.

*As antidotes for lead.*—The moist persulphuret of the hydrated peroxide of iron.

*As antidotes for arsenic.*—The moist hydrated peroxide of iron; the dry hydrated peroxide of iron; and the moist persulphuret of the hydrated peroxide of iron.

These experienced chemists add the following reflections:—This last preparation, the persulphuret of the hydrated peroxide of iron, possesses this superior advantage over all the rest, that it changes the nature of all the four poisons above noticed, and is especially applicable in those cases where we have not had time to find which of the poisons has been taken. As to the manner of administering these antidotes, and the doses which it is necessary to administer, the simplest means appear the best. The powders of zinc and iron may be suspended in any electuary, or they may be swallowed in wafer paper. The magma of the hydrated preparations of iron may be swallowed in the form of jelly, in which they are procured from the druggists. Several draughts of lukewarm water ought to follow the antidote, and the fauces be tickled with a feather, to excite vomiting and the expulsion of the poison. The efforts at vomiting scatter more effectually over the stomach the antidote which is administered. As to dose, the experiments proved that 100 grains of the powder of iron or of zinc sufficed to prevent any bad effects from 15 grains of the acetate of copper. Fifteen drachms of the moist magma of the persulphuret were required to produce the same effect with the same dose (15 grains) of the acetate of copper. To act as an antidote to 4½ grains of arsenious acid, 15 drachms of the moist magma of the persulphuret, or 30 drachms of the moist hydrated peroxide of iron, or 20 drachms of the dry hydrated peroxide of iron, were required. With regard to the time when these antidotes can be administered with advantage, in so far as the acetate of copper is concerned, the lapse of 40 minutes from the time of swallowing the poison ought not to be regarded as a sufficient reason for not administering the antidote; but arsenic is more quickly absorbed. Nevertheless, the antidote should always be administered, because, though it will not neutralize what is absorbed, it will prevent its further absorption, by decomposing what remains in the stomach.

Braithwaite from *Edin'g. Med. Times.*

